# Risk Factors for Cognitive Impairment in Patients with Type 2 Diabetes

**DOI:** 10.1155/2020/4591938

**Published:** 2020-04-23

**Authors:** Lin Sun, Xue Diao, Xiaokun Gang, You Lv, Xue Zhao, Shuo Yang, Ying Gao, Guixia Wang

**Affiliations:** ^1^Department of Endocrinology and Metabolism, The First Hospital of Jilin University, Changchun, 130021 Jilin Province, China; ^2^Department of Endocrinology and Metabolism, The University of Hong Kong-Shenzhen Hospital, Shenzhen 518000, Guangdong Province, China

## Abstract

**Objectives:**

To investigate the risk factors for cognitive impairment in Chinese type 2 diabetes mellitus (T2DM) patients of advanced age and to identify effective biomarkers of mild cognitive impairment (MCI) in these patients.

**Methods:**

Chinese T2DM patients (*n* = 120) aged 50–70 years were divided into groups with impaired (mild, moderate, and severe) and normal cognitive function based on Montreal Cognitive Assessment and Mini-Mental State Examination scores. Data regarding demographic characteristics, clinical features of diabetes, biochemical markers, and metabolomics were collected.

**Results:**

Age, educational level, duration of diabetes, fasting blood glucose (FBG), HbA1c, total cholesterol (TC), triglyceride (TG), and 24-hour urine protein were significantly associated with cognitive impairment in T2DM patients of advanced age. The severity of fundus retinopathy and the incidence of macrovascular disease also differed significantly among the groups (*P* < 0.05). Metabolomics analysis suggested that increased levels of glutamate (Glu), phenylalanine (Phe), tyrosine (Tyr), proline (Pro), and homocysteine (Hcy) and a decreased level of glutamine (Gln) were significantly associated with cognitive impairment in the T2DM patients (*P* < 0.05). Receiver operating characteristic curve analysis demonstrated that Glu, Gln, Phe, and Pro levels were significant predictors of cognitive impairment in the T2DM patients.

**Conclusions:**

Age, educational level, duration of diabetes, and the levels of FBG, HbA1c, TC, TG, and 24-hour urine protein were considered as independent risk factors for cognitive impairment in older T2DM patients. Macrovascular and microvascular diseases also were closely associated with cognitive impairment in these patients. Together, Glu and Gln levels may represent a good predictive biomarker for the early diagnosis of cognitive impairment in T2DM patients.

## 1. Introduction

Type 2 diabetes mellitus (T2DM) is characterized by relative insulin deficiency and insulin resistance, and obesity and sedentary lifestyle are generally considered to be the major risk factors [1]. According to changes in socioeconomic factors and increased practice of unhealthy lifestyle habits, the prevalence of diabetes is increasing in developing and developed countries [2]. T2DM is associated with cognitive decline, and patients with diabetes the patients exhibit worse cognitive ability and more abnormalities on brain imaging than individuals without diabetes [3, 4]. The prevalence of mild cognitive impairment (MCI) is particularly higher in T2DM patients older than 65 years [5]. Multiple long-term epidemiological studies have implicated T2DM as a risk factor for cognitive dysfunction and dementia in the elderly [6, 7].

The mechanisms of cognitive function decline and brain structural abnormalities in T2DM patients remain incompletely understood. However, research has identified particular risk factors that promote the occurrence of MCI in T2DM patients, including vascular risk factors, macrovascular diseases, microvascular complications, poor glycemic control, hyperinsulinemia, increased oxidative stress, accumulation of amyloid-beta peptide and tau hyperphosphorylation, and nerve growth factor deficiency [3, 7, 8]. Currently, there are no specific measures for preventing or treating cognitive deficits in diabetic patients, and the importance of such impairment often receives less attention than other complications of T2DM [9]. Given that interventions for cognitive impairment are reasonably effective when applied during the early stages [10], it is important to clarify the characteristics of MCI in T2DM patients and to identify the effective diagnostic markers of MCI in these patients.

In this cross-sectional study, we used the Montreal Cognitive Assessment (MoCA) and Mini-Mental State Exam (MMSE) to assess the cognitive function of T2DM patients aged 50–70 years. We aimed at determining the characteristics of cognitive impairment in T2DM patients in this age range as well as identifying potential risk factors and biomarkers from among the demographic and clinical characteristics of the patients included in this study. This information can support strategies for the early diagnosis of MCI in T2DM patients.

## 2. Materials and Methods

### 2.1. Patients and Study Design

The present study included 120 patients who were admitted to the Department of Endocrinology of the 1st Hospital of Jilin University between October 2017 and September 2018, according to in-hospital records stored in electronic databases.

The inclusion criteria for subjects were as follows: (1) age 50–70 years and (2) diagnosis of T2DM at least 3 years prior to enrollment according to the criteria of the Type 2 Diabetes Mellitus Prevention Guideline in China. The exclusion criteria were as follows: (1) acute cerebral illness within the previous 3 months; (2) significant sequel of previous cerebrovascular disease; (3) psychosis, Parkinson's disease, brain tumor, encephalitis, or epilepsy; (4) thyroid dysfunction, CO poisoning, syphilis, or other systemic diseases that could cause cognitive impairment; (5) alcohol dependence or drug abuse; (6) obvious anxiety and depression; and (7) a history of severe infection or acute diabetic complications.

### 2.2. Collection of Clinical Data

For all of the patients, we collected data for demographic and clinical characteristics, including age, gender, education level, body mass index (BMI), duration of T2DM, and history of smoking, alcohol drinking, and hypertension. Systolic blood pressure (SBP), diastolic blood pressure (DBP), and levels of fasting blood glucose (FBG), fasting C-peptide (FCP), urea nitrogen, glycosylated hemoglobin (HbA1c), total cholesterol (TC), triglyceride (TG), low-density cholesterol (LDL-C), and high-density cholesterol (HDL-C) were measured. Data related to diabetic complications such as optic fundi (I-VI phase), 24-hour urine protein, and carotid ultrasonography (collected by the Color Doppler Department) also were collected. Blood samples were obtained after fasting and analyzed by the Department of Clinical Laboratory of the First Hospital of Jilin University. The HbA1c levels were determined using an Automatic Glycohemoglobin Analyzer, and the FBG, creatinine (CRE), blood urea nitrogen (BUN), LDL-C, HDL-C, TG, and TC levels were measured by an Automatic Biochemistry Analyzer Hitachi 7060C.

BMI was calculated as body weight (kg) divided by the square of height (m^2^).

### 2.3. Cognitive Testing

The MoCA scale and MMSE scale were applied to assess the neuropsychological situation of all participants. We selected the MoCA Chinese version, which is a one-page, 10-minute, 30-point screening test to identify individuals with MCI. It includes the testing of visual space and executive functions, naming, memory, attention, language, abstract thinking, calculation, and orientation. A MoCA score ≥ 26 indicates normal cognition.

The Chinese version of the MMSE is a 30-point questionnaire used clinically to measure cognitive impairment. Any score < 27 indicates decreased cognitive function. The raw score should be corrected for educational attainment as follows: illiteracy ≤ 17 points, primary school level ≤ 20 points, secondary school level (including technical secondary school) ≤22 points, and university degree (including junior college) ≤23 points.

The 120 included patients were divided into four groups based on the MoCA and MMSE results: a normal cognitive function (NCF) group (*n* = 40, MoCA ≥ 26, and MMSE ≥ 27), a mild cognitive impairment (MCI) group (*n* = 37, MoCA < 26, and 21 ≤ MMSE ≤ 26), a moderate cognitive impairment (MoCI) group (*n* = 31, MoCA < 26, and 10 ≤ MMSE ≤ 20), and a severe cognitive impairment (SCI) group (*n* = 12, MoCA < 26, and 0 ≤ MMSE ≤ 9).

### 2.4. Metabolomics Analysis

All patients fasted for 8 hours before the collection of venous blood samples in the early morning of the next day.

The following chemical reagents were used: acetonitrile (HPLC grade, Thermo Fisher), pure water (Thermo Fisher), 1-butanol (Sigma-Aldrich), acetyl chloride (Sigma-Aldrich), and NSK-A and NSK-B isotope internal standards (Cambridge). All standards were mixed and dissolved in 2 mL pure methanol for storage at 4°C. A working solution was obtained by 100× dilution and used to extract metabolites. Amino acid and carnitine QC standards were purchased from Chromsystems.

For sample processing, a 3 mm diameter circle was punched from each dry paper blood spot and placed in a well of a Millipore multilayer 96-well plate (Millipore Corp, Billerica, MA, USA) for metabolite extraction. Then, 100 *μ*L of the working solution was added to each well. The 96-well plate was centrifuged at 1500 g for 2 minutes after gentle shaking for 20 minutes, and the filtrates were collected from the lower layer of the wells. Four blank wells are randomly selected in each plate, and in them, two low controls and two high controls were included individually. The quality control samples and filtrate samples were blown dry in pure nitrogen at 50°C. The dry samples were derivatized in a mixture of 60 *μ*L acetyl chloride and 1-butanol (1 : 9 by volume) at 65°C for 20 minutes. The derived samples were dried, and each dried sample was reconstituted in 100 *μ*L of fresh mobile phase solution for metabolite analysis.

For the detection of metabolomics markers, direct injection mass spectrometric analysis was performed using the AB Sciex 4000 QTrap system (AB Sciex, Framingham, MA, USA). The ion source of the instrument is an electrospray ion source that scans all analytes in positive ion mode. The injection volume was 20 *μ*L, the mobile phase was 80% aqueous acetonitrile, and the initial flow rate was 0.2 mL/min. Then, the flow rate was reduced to 0.1 mL/min in 0.08 min, kept constant for 1.5 min, returned to 0.2 mL/min in 0.01 min, and then maintained for 0.5 min. The ion spray voltage was 4.5 kV, the curtain air pressure was set to 20 psi, the ion source gas 1 and gas 2 were 35 psi, and the auxiliary heating gas temperature was 350°C. The system was controlled, and data were collected by the Analyst v1.6.0 software (AB Sciex, Framingham, MA, USA). Data were processed using ChemoView 2.0.2 (AB Sciex, Framingham, MA, USA).

### 2.5. Statistical Analysis

Statistical analysis was performed using SPSS 18.0 software. Mean ± standard deviation values were used to describe quantitative data if normally distributed, and the median (quartile), i.e., M (P25-P75), were used to describe the quantitative data if not normally distributed. For quantitative data with a normal distribution and homogeneity of variance, differences between groups were analyzed by one-way analysis of variance (ANOVA). To compare data with a nonnormal distribution or with homogeneity of variance, a nonparametric test was used. Multivariate logistic regression models were used to analyze risk factors associated with cognitive impairment in patients with T2DM. *P* < 0.05 denoted statistical significance.

## 3. Results

### 3.1. Demographic and Clinical Characteristics

The demographic and clinical data of the patients are presented in Table 1. The 120 enrolled patients included 82 males (68.3%) and 38 females (31.7%). The average age of the patients was 64.43 ± 15.29 years. Seventy-five patients (62.5%) had a history of hypertension, and 64 patients (53.3%) had a history of smoking.

Among the four groups with differing levels of cognitive function, statistically significant differences were found in age, duration of diabetes, BMI, education level, occurrence of cardiovascular events, smoking, FBG, HbA1c, TC, TG, and 24-hour urine protein (all *P* < 0.05).

### 3.2. Binary Logistic Regression Analysis of Baseline Data and MoCA Score

Binary logistic regression analysis was performed with age, duration of diabetes, SBP, DBP, BMI, educational level, occurrence of cardiovascular events, smoking, FBG, HbA1c, TC, TG, CRE, BUN, LDL-C, HDL-C, Homeostatic Model of Insulin Resistance (HOMA-IR), and 24-hour urine protein as independent variables and the incidence of cognitive impairment as the dependent variable. From this analysis, age, duration of diabetes, BMI, education, cardiovascular events, smoking, FBG, HbA1c, TC, TG, and 24-hour urine protein differed significantly between T2DM patients with cognitive impairment and those with normal cognitive function (Table 2, *P* < 0.05).

### 3.3. Correlation between Baseline Characteristics and Cognitive Impairment in T2DM Patients

From the results of the binary logistic regression analysis, for the ordinal multivariate logistic regression analysis, we set age, diabetes duration, BMI, education level, occurrence of cardiovascular events, smoking, FBG, HbA1c, TC, TG, and 24-hour urine protein as independent variables and the incidence of cognitive impairment as the dependent variable. The results showed that the following factors were independently associated with cognitive impairment in T2DM patients: age, educational level, duration of diabetes, FBG, HbA1c, TC, TG, and 24-hour urine protein (Table 3).

#### 3.3.1. Correlation between Fundus Retinopathy and Cognitive Impairment in T2DM

Significant differences in the degree of fundus retinopathy were observed between the four groups (*P* < 0.05; Table 4).

#### 3.3.2. Correlation between Macrovascular Diseases and Cognitive Impairment in T2DM

Significant differences in the severity of macrovascular diseases were observed between the four groups of T2DM patients (*P* < 0.05; Table 5).

### 3.4. Correlation between Metabolomics Indicators and Cognitive Impairment in T2DM

**Figure 1:**
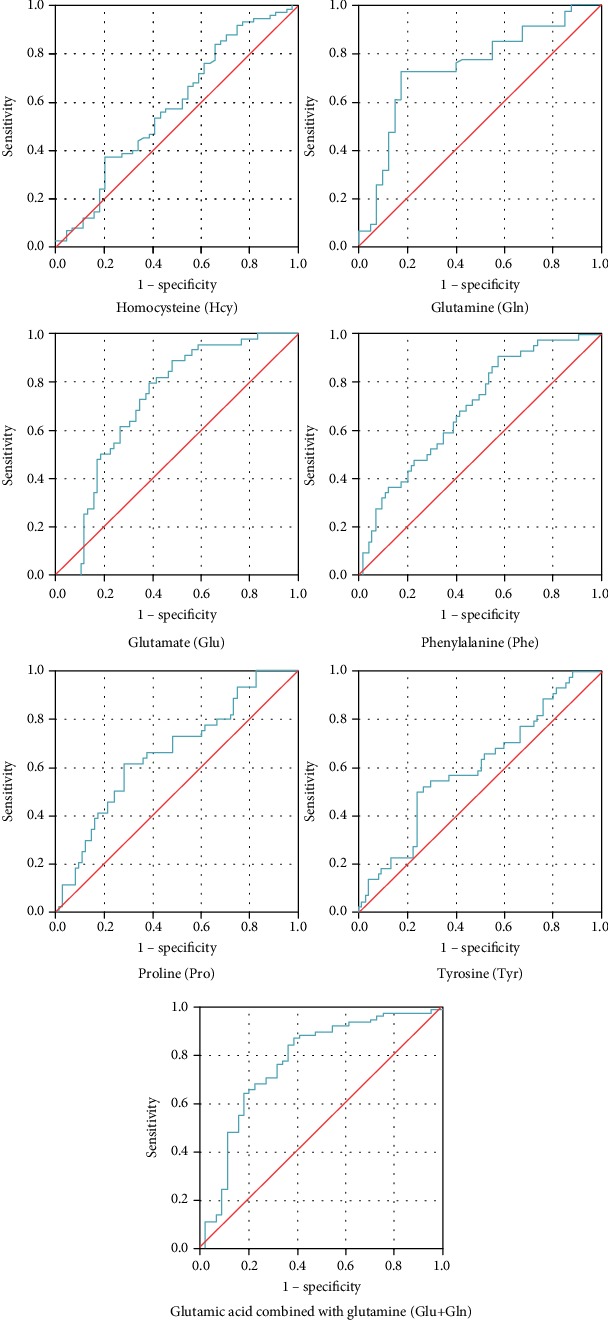
ROC curve analysis of the association of amino acid levels with cognitive impairment in T2DM patients of advanced age.

The results for metabolomics indicators in all patients showed that glutamate (Glu), glutamine (Gln), phenylalanine (Phe), tyrosine (Tyr), proline (Pro), and homocysteine (Hcy) levels were statistically different between the four groups (*P* < 0.05). Of those, Glu, Phe, Tyr, Pro, and Hcy levels increased with the development of cognitive impairments, whereas the Gln level decreased (Table 6). The results of receiver operating characteristic (ROC) curve analysis (Figure 1) of the six amino acids suggested that Glu, Gln, Phe, and Pro levels may be meaningful in the diagnosis of cognitive dysfunction in patients with T2DM (Table 7). Combined analysis of Glu and Gln levels by ROC curve analysis suggested that a diagnosis made based on Glu and Gln is more meaningful for the early diagnosis of cognitive impairments in T2DM patients. Normal ranges for clinical and metabolomics indicators are shown in Table A1 in appendix materials. Correlation between the level of acylcarnitine and cognitive impairment in T2DM patients of advanced age was shown in Table A2.

## 4. Discussion

Greater cognitive decline occurs among older people (>50 years of age) with T2DM than among the general nondiabetic population of the same age. The results of our present study show that the severity of cognitive impairment in these T2DM patients was closely related to age and education level. As T2DM patients age, brain atrophy and lacunar infarction are observed with greater frequency. In addition, T2DM patients may exhibit small and punctate white-matter lesions, decreased brain volume, altered vascular function, oxidative stress, and accumulation of glycation end products. Biessels et al. [3] summarized the relationship between cognitive decline and age in T2DM patients and determined that cognitive impairment in T2DM patients occurs mostly from the ages of 40–80 years, and particularly from 60–80 years. The available evidence suggests a negative correlation between education and the incidence of dementia [5]. A potential explanation is that individuals with a higher education level and knowledge workers have a higher synaptic density in the cortex, which increases the brain's storage capacity, and thus, the symptoms of dementia are delayed for 4–5 years.

As the duration of T2DM increases, macrovascular and microvascular diseases, oxidative stress damage, and insulin resistance are likely to increase the damage to neurons. Logroscino et al. [11] found that cognitive decline in T2DM patients was positively correlated with the duration of diabetes, and the risk of cognitive impairment was significantly increased in patients who had been diagnosed with T2DM more than 15 years previously. Studies have shown that with the development of diabetes, image memory and graphic cognitive ability decline [12]. The results of the present study also confirm these phenomena. For example, the degree of carotid stenosis was positively correlated with the severity of cognitive dysfunction in our patients. Diabetes accelerates vascular aging, reduces cerebral blood flow, causes focal ischemic infarction, leads to diffusion of white matter and basal ganglia, and causes neuronal damage and apoptosis, leading to impaired executive cognitive function [5, 13]. On the other hand, diabetes can affect microvascular endothelial cell function, leading to the impairment of the blood–brain barrier and neuroinflammatory reactions. Studies have shown that neurovascular units are related to the dynamics of blood flow in the brain. Changes in microvascular structure, decreased numbers of capillaries, and increased arteriovenous shorts in T2DM patients may affect the transport of nutrients to nerve tissue, and brain tissues are easily damaged by oxygen deficiency when perfusion pressure or blood flow is decreased [13]. Consistently, our data revealed a positive correlation between the staging of fundus retinopathy and the severity of cognitive dysfunction.

Our findings agree with those of other studies showing that hyperglycemia, as accessed by HbA1c concentration, is correlated with cognitive dysfunction in T2DM patients. Long-term hyperglycemia may lead to the thickening of the cerebral vascular muscle basement membrane, reduce cerebral blood circulation, and directly damage neurons. Studies have confirmed that reduced cerebral blood flow obstructs the brain's ability to understand, process, integrate information, etc., ultimately leading to impaired learning and memory ability [14]. One proposed mechanism involves fibronectin (Fn), which is a macromolecular glycoprotein found in plasma and the extracellular matrix that is damaged during periods of hyperglycemia. This damage causes the capillaries within the brain to proliferate, increasing the permeability of the blood–cerebrospinal fluid barrier. More inflammatory cell molecules and antibodies are then allowed to attack brain cells through the blood–cerebrospinal fluid barrier, leading to a decline in cognitive function [15]. In addition, during hyperglycemia, tau protein and amyloid *β* are nonenzymatically glycosylated to form glycosylation end products (AGEs), which are known to participate in the pathological manifestations of Alzheimer's disease.

The present study also identified high levels of TC and TG as risk factors for the development of cognitive impairment in older T2DM patients. T2DM is commonly complicated by lipid metabolism disorder, and abnormal lipid metabolism is also closely related to neurological diseases such as Alzheimer's disease, epilepsy, and Parkinson's disease. Elevated serum TC and TG levels can cause damage to brain vascular endothelial cells and may interfere with the metabolism of amyloid precursor proteins, thereby, accelerating the production and accumulation of A*β* and leading to cognitive dysfunction [16]. Farr et al. [16] proposed that increased TC may affect amino acid receptors, impairing hippocampal synaptic transmission and affecting memory formation. Frias et al. [17] found that increased TG was related to impaired speech knowledge. These studies found that higher TC levels correlated with more severe cognitive impairment.

Amino acid metabolism also directly affects the activity of the nervous system. For example, it was reported that the glutamate level is associated with the level of cognition. Abnormal glutamate metabolism and glutamate receptor function are associated with a variety of neurological diseases including Alzheimer's disease/amnestic mild cognitive impairment (aMCI) [18]. In addition, glutamate production will increase compensatorily to protect the cognitive function in patients with cognitive impairment. The results of our metabolomics analysis showed that Glu, Phe, Tyr, Pro, and Hcy levels increased with the development of cognitive impairment, while the Gln level decreased. Proline is a metabolite of glutamate. The upregulation of proline may be related to abnormal protein aggregation in the brain and abnormal glutamate-proline metabolic signaling [19]. Tyrosine, phenylalanine, and phenethylamine are sulfur-containing amino acids or related products involved in the metabolism of various neurotransmitters. When cognitive impairment begins to occur, there may also be compensatory mechanisms that promote the upregulation of these neurotransmitter precursors to supplement the lack of neurotransmitters in the brain [20]. Many reports have indicated that hyperhomocysteinemia is common in patients with T2DM. This may be due to the lack of insulin in T2DM patients, which affects the catabolism of homocysteine. Moreover, hyperhomocysteinemia may be a risk factor for cognitive decline [20].

Additionally, it is notable that the level of Gln was negatively correlated with cognitive dysfunction in our statistical analysis, and we believe this may be related to the course of diabetes. Studies have shown that glutamate metabolism is closely related to insulin resistance. Tulipani et al. [21] found a significant increase in the glutamate level in 64 patients with morbid obesity and early-stage diabetes, which was proportional to the insulin resistance index in these patients. Takashina et al. [22] also showed that the glutamate level was increased while the glutamine level was decreased in 83 obese patients. Concomitantly, HOMA-IR and HOMA-*β* were directly proportional to glutamate levels and inversely proportional to glutamine levels. In animal studies, Perdigon et al. [23] observed a decline in cognitive function in high-fat diet-fed rats and a concomitant decline in glutamate levels.

Diabetes is closely associated with the occurrence of cognitive dysfunction or dementia. However, there is no unified diagnostic standard for diabetic cognitive dysfunction currently. Diabetes-related cognitive disorders are often overlooked in the diagnosis and treatment process, resulting in many diabetic patients with cognitive impairment suffering from diminishing memory for a long time [24–26]. Our study analyzed the data from 120 diabetic patients and confirmed some clinical features and risk factors related to cognitive decline. In addition, according to existing reports, there are many risk factors for cognitive decline in type 2 diabetes patients, including diabetes-specific factors, demographic factors, genetic factors, and lifestyle factors. Nevertheless, there has been no clinical research discussing the effect of amino acid metabolism on the process of diabetes cognitive dysfunction. Amino acids, as the basic units of protein, participate in the synthesis and metabolism of various active substances in the body and play important roles in learning, memory, and nerve conduction. This study examined cognition-related amino acid metabolites in 120 diabetic patients, and such research is very important for the discovery of valuable biomarkers for early diagnosis of diabetes-related cognitive impairment.

The present study has a number of limitations that should be considered. First, this was a cross-sectional study with a small sample size, which may limit the reliability of the results. Next, we did not exclude individuals who had recently received medicines that could affect cognitive function. Furthermore, we did not collect data related to daily exercise, which has been shown to strongly affect the level of cognitive impairment. Additionally, we did not include food recall in our questionnaire because of inconvenient operation, and any changes in food intake can have a considerable influence on the cognitive index. Thus, this is a limitation in our research design as well. Finally, we did not investigate the influence of hypoglycemia on the cognitive function of T2DM patients. Current research results are controversial, and due to the recall bias introduced by patients' ability to recall of hypoglycemic events, we did not collect information regarding hypoglycemia.

## 5. Conclusion

In summary, age, educational level, duration of diabetes, and levels of FBG, HbA1c, TC, TG, and 24-hour urine protein were identified as independent risk factors for cognitive impairment in T2DM patients of advanced age (50–70 years). Macrovascular and microvascular diseases also were strongly associated with cognitive decline in these patients. The levels of glutamate, glutamine, proline, and phenylalanine may be good predictive biomarkers of cognitive impairment in T2DM patients of advanced age, and together, glutamate and glutamine levels may represent the most effective biomarker panel among the identified amino acids. This finding could have an important clinical impact on the search for predictive serological markers for early diagnosis of cognitive impairment in T2DM patients. However, further investigation of the value of these predictors in practical application is warranted.

## Figures and Tables

**Table 1:** Demographic and clinical characteristics of the 120 T2DM patients grouped according to the degree of cognitive impairment.

Characteristics	NCF group (*n* = 40)	MCI group (*n* = 37)	MoCI group (*n* = 31)	SCI group (*n* = 12)	*F*/*χ*^2^	*P* value
Age (y)	53.01 ± 2.43	56.45 ± 4.62	59.54 ± 3.16	64.45 ± 5.24	11.243	0.012^∗^
Duration of diabetes (y)	5.12 ± 1.13	6.34 ± 2.53	8.13 ± 2.16	10.14 ± 8.24	14.35	0.008^∗^
Smoking *n* (%)	15 (37.50%)	18 (48.65%)	23 (74.19%)	9 (75.00%)	8.532	0.018^∗^
Education level (y)	33 (82.50%)	24 (64.86%)	12 (38.71%)	4 (33.33%)	9.135	0.013^∗^
Cardiovascular event	13 (32.50%)	15 (40.54%)	16 (51.61%)	7 (58.33%)	7.357	0.042^∗^
TC (mmol/L)	3.42 (3.34, 4.32)	3.33 (3.24, 4.56)	3.84 (3.12, 4.78)	4.51 (3.56, 5.78)	12.505	0.002^∗^
TG (mmol/L)	2.51 (1.23, 3.21)	2.71 (1.34, 3.67)	3.13 (1.56, 4.56)	4.24 (1.67, 5.32)	13.87	0.004^∗^
HDL-C (mmol/L)	0.83 ± 0.58	0.85 ± 0.78	0.94 ± 0.67	1.14 ± 0.78	2.201	0.117
LDL-C (mmol/L)	2.13 ± 0.86	2.43 ± 0.97	2.84 ± 0.87	3.32 ± 1.12	2.35	0.102
BMI (kg/m^2^)	23.2 ± 1.5	24.1 ± 1.8	25.2 ± 2.1	26.7 ± 2.4	1.824	0.048^∗^
SBP (mmHg)	124 ± 21	137 ± 23	135 ± 19	142 ± 21	3.955	0.138
DBP (mmHg)	78 ± 9	82 ± 10	79 ± 8	84 ± 11	2.538	0.281
FBG (mmol/L)	6.84 ± 1.75	6.93 ± 2.25	7.43 ± 2.16	8.83 ± 2.36	10.266	0.044^∗^
HbA1c (mmol/L)	6.7 ± 1.2	6.9 ± 1.4	7.3 ± 1.8	7.7 ± 1.9	12.551	0.002^∗^
AST (U/L)	31.55 (13.45, 38.54)	23.00 (18.65, 41.42)	25.70 (20.12, 43.56)			
ALT (U/L)	21.2 (10.34, 25.67)	43.70 (32.45, 48.64)	2.239	0.113	2.926	0.134
ALP (U/L)	72.75 (50.46, 89.44)	71.45 (43.45, 89.34)	81.34 (68.34, 99.34)	85.34 (72.39, 91.77)	2.509	0.285
Prealbumin (g/L)	0.26 (0.21, 0.43)	0.27 (0.22, 0.34)	0.21 (0.15, 0.29)	0.25 (0.17, 0.23)	4.982	0.544
Apo-A1 (g/L)	0.93 (0.67, 1.31)	0.95 (0.71, 1.23)	1.05 (0.73, 1.22)	1.03 (0.75, 1.32)	3.456	0.436
Apo-B (g/L)	0.96 (0.94, 1.03)	0.98 (0.95, 1.04)	0.83 (0.78, 0.88)	0.92 (0.85, 0.97)	2.456	0.754
T Bil (*μ*mol/L)	13.06 ± 6.12	11.45 ± 5.38	14.42 ± 7.18	13.45 ± 6.10	1.345	0.435
I Bil (*μ*mol/L)	10.41 ± 3.24	7.63 ± 2.42	8.91 ± 3.24	6.93 ± 3.21	2.452	0.654
D Bil (*μ*mol/L)	3.42 (3.21, 3.56)	2.91 (2.34, 3.56)	3.31 (2.67, 3.97)	2.54 (2.14, 3.08)	5.356	0.267
TBA (*μ*mol/L)	3.41 ± 1.34	4.42 ± 2.05	3.56 ± 1.42	3.13 ± 1.54	1.375	0.135
WBC	6.31 (4.87, 7.46)	7.54 (5.36, 8.46)	6.04 (5.03, 7.98)	7.04 (5.35, 9.75)	3.467	0.079
NE%	0.60 (0.45, 0.71)	0.51 (0.43, 0.76)	0.56 (0.51, 0.68)	0.62 (0.55, 0.73)	2.349	0.275
LY%	0.33 (0.25, 0.38)	0.31 (0.27, 0.39)	0.37 (0.33, 0.41)	0.41 (0.36, 0.49)	0.923	0.319
24 h UP (g/24 h)	0.21 ± 0.07	0.26 ± 0.05	0.24 ± 0.02	0.41 ± 0.05	15.563	0.014^∗^
24 h mAlb (mg/24 h)	43.82 ± 30.34	56.36 ± 40.23	55.23 ± 43.54	65.34 ± 46.32	3.561	0.145
CRP (mg/L)	3.35 (2.34, 5.42)	4.36 (3.45, 5.78)	3.22 (2.45, 4.67)	3.52 (2.67, 4.66)	2.345	0.542
SCr (*μ*mol/L)	72.6 (63.46, 77.54)	77.56 (72.45, 79.54)	63.42 (54.67, 69.13)	73.34 (65.88, 78.64)	1.386	0.638
BUN (mmol/L)	5.23 (4.24, 6.75)	6.21 (5.78, 6.94)	5.61 (4.35, 7.68)	5.11 (4.24, 6.97)	2.457	0.846
UA (*μ*mol/L)	245.32 ± 53.45	342.54 ± 43.53	354.65 ± 34.67	367.34 ± 64.45	8.642	0.063
Alb (g/L)	38.21 (33.56, 41.34)	40.41 (35.78, 43.67)	39.52 (32.56, 42.87)	35.64 (31.56, 40.23)	5.682	0.105
Glb (g/L)	28.23 (25.78, 30.14)	28.23 (23.56, 32.67)	23.54 (21.38, 27.58)	25.45 (22.46, 26.95)	2.843	0.638
CO_2_CP (mmol/L)	25.62 (23.55, 27.54)	25.33 (21.45, 29.45)	26.29 (24.56, 29.76)	27.23 (25.32, 30.12)	0.468	0.462
CHE (U/L)	9456.37 ± 2539.60	9228.10 ± 2134.96	9153.15 ± 2671.12	9423.19 ± 2751.13	0.246	0.723

^∗^ *P* < 0.05. Abbreviations: TC: total cholesterol; TG: triglyceride; HDL-C: high-density lipoprotein cholesterol; LDL-C: low-density cholesterol.

**Table 2:** Binary logistic regression analysis of associations between baseline characteristics and MoCA score in T2DM patients of advanced age.

	Regression coefficient (*β*)	Standard error (SE)	Wald	*P*	Odds ratio (OR)	95% confidence interval (CI)
Age duration of diabetes (y)	1.177	1.032	2.123	0.002^∗^	1.245	1.132-3.236
	1.321	2.143	2.876	0.025^∗^	1.432	1.232-5.441
BMI (kg/m^2^)	1.564	1.342	5.4 2	0.042^∗^	1.134	1.152-3.221
SBP (mmHg)	0.464	1.302	2.434	0.221	1.589	0.878-2.875
DBP (mmHg)	0.267	2.221	0.014	0.412	1.025	0.653-2.624
Education level (y)	6.267	1.221	12.014	0.004^∗^	2.025	0.953-3.624
Cardiovascular event	0.836	0.487	3.172	0.033^∗^	1.122	0.162-4.095
Smoking (%)	0.369	0.115	5.647	0.039^∗^	1.463	1.161-5.835
FBG (mmol/L)	1.421	0.313	2.721	0.021^∗^	1.224	0.851-2.845
HbA1c (mmol/L)	2.932	0.209	0.296	0.014^∗^	1.325	1.143-4.774
SCr (*μ*mol/L)	0.232	5.578	1.683	0.732	4.174	1.435-7.456
BUN (mmol/L)	0.332	6.578	2.683	0.932	1.274	0.735-5.456
TC (mmol/L)	2.345	0.421	2.532	0.027^∗^	5.453	0.257-3.052
TG (mmol/L)	1.622	0.426	4,643	0.035^∗^	2.445	0.561-1.625
LDL-C (mmol/L)	0.433	1.234	3.567	0.061	1.164	0.868-2.567
HDL-C (mmol/L)	0.632	1.578	3.683	0.532	2.574	0.435-1.456
HOMA-IR	0.142	1.765	4.643	0.417	4.245	0.153-2.345
24 h UP (g/24 h)	3.213	4.789	5.890	0.027^∗^	2.865	0.346-7.546

^∗^ *P* < 0.05.

**Table 3:** Analysis of risk factors for cognitive impairment in T2DM patients of advanced age.

	Regression coefficient (*β*)	Standard error (SE)	Wald	*P*	Odds ratio (OR)	95% confidence interval (CI)
Age (y)	5.177	0.032	2.123	0.002^∗^	4.253	1.132-3.236
Duration of diabetes (y)	3.321	0.143	2.876	0.025^∗^	1.424	1.232-5.441
Smoking (%)	2.369	1.115	10.647	0.059	3.245	1.161-1.835
BMI (kg/m^2^)	1.564	0.342	5.421	0.052	0.934	1.152-1.221
Cardiovascular event	-0.836	0.487	3.172	0.073	43.567	0.162-1.095
Education level (y)	5.267	1.221	7.014	0.012^∗^	5.356	0.453-2.637
FBG (mmol/L)	4.421	0.313	2.721	0.021^∗^	6.435	0.851-2.845
HbA1c (mmol/L)	6.032	0.209	0.296	0.014^∗^	1.956	1.143-4.774
TC (mmol/L)	2.345	0.421	2.532	0.027^∗^	1.345	0.257-3.052
TG (mmol/L)	1.622	0.426	4,643	0.035^∗^	1.643	0.561-1.625
24 h UP (g/24 h)	2.134	1.567	1.754	0.041^∗^	3.175	0.386-6.424
HOMA-IR	0.142	1.765	4.643	0.417	4.245	0.153-2.345

^∗^ *P* < 0.05.

**Table 4:** Correlation between fundus retinopathy and cognitive impairment in T2DM patients of advanced age.

Retinopathy	NCF group (*n* = 40)	MCI group (*n* = 37)	MoCI group (*n* = 31)	SCI group (*n* = 12)	*χ* ^2^	*P* value
Mild degree (phase I-II)	39	35	28	7	16.194	0.002^∗^
Severe degree (phase III-IV)	1	2	3	5		

^∗^ *P* < 0.05.

**Table 5:** Correlation between macrovascular disease and cognitive impairment in T2DM patients of advanced age.

Carotid ultrasound	NCF group (*n* = 40)	MCI group (*n* = 37)	MoCI group (*n* = 31)	SCI group (*n* = 12)	*χ* ^2^	*P* value
Mild stenosis	36	33	22	6	14.437	0.003^∗^
Severe stenosis	4	4	9	6		

^∗^ *P* < 0.05.

**Table 6:** Correlations between amino acid levels (*μ*mol/L) and cognitive impairment in T2DM patients of advanced age.

Amino Acid	NCF group (*n* = 40)	MCI group (*n* = 37)	MoCI group (*n* = 31)	SCI group (*n* = 12)	*F*	*P* value
Ala	135.84 (111.22, 156.64)	134.51 (125.86, 153,81)	133.43 (105.13, 149.17)	126.26 (100.07, 175.67)	1.345	0.862
Arg	4.19 (2.23, 7.30)	3.04 (2.23, 4.13)	2.92 (2.05, 3.84)	1.10 (0.85, 3.29)	2.342	0.013
Asn	78.30 ± 15.78	75.76 ± 19.57	75.60 ± 20.34	73.00 ± 20.13	0.284	0.837
Asp	27.33 (18.52, 36.89)	18.74 (14.17, 22.60)	16.40 (12.80, 18.21)	14.26 (13.37, 17.08)	2.920	0.097
Cit	20.11 (17.94, 23.32)	22.17 (18.26, 25.14)	20.59 (17.70, 22.58)	16.96 (14.89, 19.18)	1.585	0.167
Orn	12.39 (10.15, 15,39)	9.30 (8.67, 11.06)	9.05 (7.53, 10.35)	10.11 (7.25, 9.48)	2.447	0.061
Gln	2.33 (7.35, 14.09)	4.93 (5.75, 6.54)	6.01 (4.59, 5.37)	9.02 (2.04, 4.11)	6.678	0.001^∗^
Lys	165.79 (130.53, 259.59)	106.48 (101.65, 115.36)	86.26 (80.31, 93.99)	40.89 (35.79, 72.03)	3.275	0.001
Met	18.87 (16.54, 24.10)	16.52 (14.85, 19.37)	16.21 (13.84, 18.16)	17.52 (14.86, 18.94)	2.179	0.061
His	50.67 (42.06, 190.74)	34.99 (32.94, 38.69)	43.58 (29.07, 37.23)	33.66 (26.58, 33.79)	3.111	0.072
Leu	160.23 (138.89, 189.05)	134.87 (109.26, 152.48)	130.26 (115.28, 146.71)	143.43 (111.91, 152.58)	1.424	0.067
Gly	172.83 (160.11, 196.85)	166.34 (147.75, 178.87)	162.65 (153.88, 176.33)	171.11 (146.03, 184.29)	2.532	0.087
Glu	213.57 (117.93, 140.35)	184.93 (101.56, 123.83)	132.12 (92.05, 128.00)	108.34 (108.98, 250.08)	4.256	0.002^∗^
Trp	45.96 (41.89, 54.74)	42.86 (35.50, 51.09)	40.58 (35.37, 47.35)	55.25 (40.61, 67.82)	2.245	0.083
Pro	608.33 (506.93, 739.37)	532.34 (481.76, 688.28)	521.25 (421.82, 586.46)	529.97 (323.26, 457.34)	5.920	0.044^∗^
Phe	36.79 (32.60, 43.96)	34.17 (28.30, 36.24)	32.01 (28.96, 36.99)	31.69 (27.87, 33.40)	6.645	0.003^∗^
Pip	400.36 ± 98.18	368.17 ± 58.63	377.95 ± 59.50	364.14 ± 51.14	4.384	0.076
Tyr	52.02 ± 10.64	49.02 ± 9.50	46.29 ± 12.18	41.32 ± 8.44	6.420	0.020^∗^
Val	154.22 ± 23.46	144.72 ± 30.18	143.20 ± 28.37	147.70 ± 15.14	1.142	0.335
Thr	52.02 ± 10.64	45.02 ± 9.50	46.29 ± 12.10	46.32 ± 8.44	2.920	0.057
Ser	40.93 ± 6.67	42.45 ± 6.55	38.63 ± 7.25	44.20 ± 7.92	2.447	0.067
Hcy	11.54 ± 1.35	10.34 ± 1.23	9.75 ± 1.07	6.45 ± 1.33	5.678	0.045^∗^
Cys	2.56 ± 1.03	2.11 ± 0.98	2.64 ± 0.94	2.14 ± 1.09	2.179	0.094
Val/Phe	4.19 (3.70, 4.55)	4.40 (3.78, 4.95)	4.28 (3.91, 4.76)	4.79 (4.50, 5.15)	3.253	0.157
Tyr/Cit	1.33 (1.08, 1.68)	1.12 (0.95, 1.34)	1.14 (0.97, 1.42)	1.47 (1.26, 1.79)	2.173	0.055
Phe/Tyr	0.75 (0.65, 0.86)	0.67 (0.63, 0.76)	0.70 (0.62, 0.81)	0.74 (0.68, 0.84)	2.179	0.258
Orn/Cit	0.57 (0.46, 0.79)	0.44 (0.41, 0.56)	0.45 (0.40, 0.52)	0.50 (0.42, 0.52)	3.119	0.051
Met/Phr	0.52 (0.46, 0.59)	0.50 (0.45, 0.56)	0.48 (0.45, 0.55)	0.57 (0.45, 0.57)	3.420	0.118
Met/Leu	0.13 (0.12, 0.14)	0.13 (0.11, 0.16)	0.12 (0.10, 0.13)	0.15 (0.11, 0.16)	1.132	0.303
Gly/Ala	1.10 (0.91, 1.35)	1.05 (0.88, 1.25)	1.13 (0.92, 1.24)	0.96 (0.88, 1.23)	2.920	0.562
Cit/Arg	5.30 (2.40, 9.06)	7.14 (5.13, 10.24)	7.09 (5.13, 10.33)	19.13 (5.52, 21.58)	1.585	0.054
Arg/Orn	0.37 (0.24, 0.44)	0.28 (0.22, 0.40)	0.32 (0.24, 0.42)	0.13 (0.11, 0.35)	2.447	0.085
Val/Phe	4.19 (3.70, 4.55)	4.40 (3.78, 4.95)	4.28 (3.91, 4.76)	4.79 (4.50, 5.15)	3.253	0.157
Tyr/Cit	1.33 (1.08, 1.68)	1.12 (0.95, 1.34)	1.14 (0.97, 1.42)	1.47 (1.26, 1.79)	2.173	0.055

^∗^ *P* < 0.05.

**Table 7:** ROC curve analysis of the ability of the levels of six amino acids to predict cognitive impairment in T2DM patients of advanced age.

Amino acid	AUC	*P* value	Cut-point	Sensitivity	Specificity
Hcy	0.581	0.142	9.500	0.840	0.341
Gln	0.739	0.001^∗^	5.902	0.722	0.825
Glu	0.721	0.002^∗^	116.431	0.795	0.613
Phe	0.697	0.003^∗^	31.746	0.909	0.427
Pro	0.661	0.004^∗^	598.183	0.614	0.719
Tyr	0.602	0.064	52.174	0.501	0.747
Gln+Glu	0.769	0.001^∗^	0.610	0.867	0.614

^∗^ *P* < 0.05.

## Data Availability

The data used to support the findings of this study are available from the corresponding author upon request.

## Supplementary Materials

Supplementary materials Table A1: reference ranges for indicators. Table A2: correlation between level of acylcarnitine (*μ*mol/L) and cognitive impairment in T2DM patients of advanced age.
