# Supplementary material for: Risk Factors for Cognitive Impairment in Patients with Type 2 Diabetes
Source: J Diabetes Res. 2020 Apr 23;2020:4591938. doi: 10.1155/2020/4591938 (PMC7196145; doi:10.1155/2020/4591938)
Supplement: Supplementary materials — Table A1: reference ranges for indicators. Table A2: correlation between level of acylcarnitine (μmol/L) and cognitive impairment in T2DM patients of advanced age. [file 4591938.f1.docx]

Appendix Table:

Table A1. Reference ranges for indicators

| Indicator (μmol/L) | Abbreviation | Lower limit of normal | Upper limit of normal |  |
| --- | --- | --- | --- | --- |
| Alanine | Ala | 50.00 | 280.00 |  |
| Alanine aminotransferase | ALT | 9 | 50 |  |
| Albumin | Alb | 40 | 55 |  |
| Alkaline phosphatase | ALP | 45 | 125 |  |
| Apolipoprotein A1 | Apo-A1 | 0.80 | 2.36 |  |
| Apolipoprotein B | Apo-B | 0.42 | 1.26 |  |
| Arginine | Arg | 0.80 | 15.00 |  |
| Asparagine | Asn | 40.00 | 120.00 |  |
| Aspartate transaminase | AST | 15.00 | 40.00 |  |
| Aspartic acid | Asp | 10.00 | 70.00 |  |
| Blood urea nitrogen | BUN | 3.10 | 8.00 |  |
| Carbon dioxide combing power | CO_2_CP | 22 | 30 |  |
| Choline esterase | CHE | 3950 | 10000 |  |
| Citrulline | Cit | 6.50 | 40.00 |  |
| Creatinine | CRE | 41.00 | 73.00 |  |
| C-reactive protein | CRP | 0 | 3.5 |  |
| Cysteine | Cys | 0.30 | 5.00 |  |
| Diastolic blood pressure | DBP | 60 | 90 |  |
| Direct bilirubin | D Bil | 0 | 6.8 |  |
| Fasting plasma glucose | FPG | 3.90 | 6.10 |  |
| Globulin | Glb | 20 | 40 |  |
| Glutamine | Gln | 2.00 | 20.00 |  |
| Glutamate | Glu | 50.00 | 250.00 |  |
| Glycine | Gly | 50.00 | 300.00 |  |
| Glycosylated hemoglobin A1c | HbA1c | 4.27% | 6.07% |  |
| Homocysteine | Hcy | 5.00 | 30.00 |  |
| Histidine | His | 10.00 | 300.00 |  |
| High-density lipoprotein cholesterol | HDL-C | 0.76 | 2.10 |  |
| Indirect bilirubin | I Bil | 5.1 | 21.4 |  |
| Leucine | Leu | 50.00 | 360.00 |  |
| Low-density lipoprotein cholesterol | LDL-C | 2.06 | 3.10 |  |
| Lysine | Lys | 20.00 | 400.00 |  |
| Methionine | Met | 8.00 | 80.00 |  |
| Ornithine | Orn | 5.00 | 50.00 |  |
| Phenylalanine | Phe | 20.00 | 80.00 |  |
| Piperamide | Pip | 60.00 | 600.00 |  |
| Proline | Pro | 180.00 | 1220.00 |  |
| Serine | Ser | 30.00 | 110.00 |  |
| Systolic blood pressure | SBP | 90 | 130 |  |
| Threonine | Thr | 10.00 | 75.00 |  |
| Total bile acid | TBA | 0 | 10 |  |
| Total bilirubin | T Bil | 0 | 26 |  |
| Total Cholesterol | TC | 2.60 | 6.00 |  |
| Triglyceride | TG | 0.26 | 1.80 |  |
| Tryptophan | Trp | 20.00 | 100.00 |  |
| Tyrosine | Tyr | 20.00 | 100.00 |  |
| Urine acid | UA | 150 | 360 |  |
| Valine | Val | 75.00 | 215.00 |  |
| White blood cells | WBC | 3.5x10^9^ | 8.5x10^9^ |  |
| Free carnitine |  | 15.00 | 55.00 |  |
| Acetylcarnitine | C2 | 2.00 | 20.00 |  |
| Propionyl carnitine | C3 | 0.30 | 3.30 |  |
| Butyryl carnitine | C4 | 0.07 | 0.50 |  |
| Hydroxybutyryl carnitine | C4-OH | 0.03 | 0.20 |  |
| Succinylcarnitine | C4DC | 0.15 | 1.50 | |
| Isovalerylcarnitine | C5 | 0.06 | 0.30 | |
| Hydroxyisovaleryl carnitine | C5-OH | 0.10 | 0.60 | |
| Glutaryl carnitine | C5DC | 0.02 | 0.22 | |
| Pentenoylcarnitine | C5:1 | 0.02 | 0.15 | |
| Hexanoyl carnitine | C6 | 0.03 | 0.20 | |
| Octanoylcarnitine | C8 | 0.03 | 0.30 | |
| Decanoylcarnitine | C10 | 0.05 | 0.50 | |
| Lauroyl carnitine | C12 | 0.04 | 0.30 | |
| Myristoyl carnitine | C14 | 0.05 | 0.30 | |
| Hydroxy myristyl carnitine | C14-OH | 0.02 | 0.12 | |
| Myristyl diacylcarnitine | C14DC | 0.01 | 0.10 | |
| Myristyl carnitine | C14:1 | 0.04 | 0.30 | |
| Palmitoylcarnitine | C16 | 0.50 | 3.00 | |
| Hydroxypalmitoyl carnitine | C16-OH | 0.02 | 0.12 | |
| Hydroxypalmitoyl carnitine | C16:1-OH | 0.02 | 0.18 | |
| Octadecylcarnitine | C18 | 0.20 | 1.50 | |
| Eicosyl carnitine | C20 | 0.02 | 0.12 | |
| Teflonyl carnitine | C22 | 0.03 | 0.16 | |
| Twenty-four acid alkaloid | C24 | 0.02 | 0.12 | |
| Hexahexyl carnitine | C26 | 0.01 | 0.08 | |

## Table A2. Correlation between level of acylcarnitine (μmol/L) and cognitive impairment in T2DM patients of advanced age.

| Acylcarnitine | NCF group (n=40) | MCI group (n=37) | MoCI group (n=31) | SCI group (n=12) | F | P value | |
| --- | --- | --- | --- | --- | --- | --- | --- |
| C4 | 0.17(0.13,0.23) | 0.18(0.14,0.23) | 0.17(0.14,0.23) | 0.19(0.14,0.21) | 2.670 | 0.123 |  |
| C3 | 1.51(1.14,1.90) | 1.29(1.12,1.79) | 1.37(0.86,1.86) | 1.09(0.75,1.52) | 3.425 | 0.210 |  |
| C0 | 33.01(27.45,41.05) | 31.32(28.68,37.43) | 38.52(28.75,43.72) | 37.12(30.03,41.39) | 2.179 | 0.407 |  |
| C8 | 0.07(0.05,0.10) | 0.06(0.04,0.09) | 0.07(0.05,0.09) | 0.06(0.05,0.06) | 3.119 | 0.309 |  |
| C2 | 10.14±2.74 | 9.43±2.52 | 9.50±3.59 | 6.82±1.89 | 3.275 | 0.054 |  |
| C16 | 0.79±0.26 | 0.72±0.28 | 0.66±0.24 | 0.70±0.28 | 1.585 | 0.197 |  |
| C14 | 0.04±0.02 | 0.03±0.02 | 0.04±0.01 | 0.03±0.01 | 3.111 | 0.059 |  |
| C5:1 | 0.04(0.03,0.06) | 0.04,0.03,0.06) | 0.03(0.02,0.05) | 0.06(0.05,0.06) | 3.420 | 0.114 |  |
| C5DC | 0.05(0.04,0.07) | 0.07(0.04,0.08) | 0.07(0.04,0.09) | 0.04(0.03,0.06) | 1.132 | 0.220 |  |
| C5-OH | 0.21(0.13,0.28) | 0.15(0.12,0.18) | 0.17(0.11,0.19) | 0.15(0.14,0.35) | 2.920 | 0.061 |  |
| C5 | 0.11(0.83,0.16) | 0.11(0.08,0.13) | 0.11(0.09,0.13) | 0.92(0.86,0.14) | 1.585 | 0.673 |  |
| C4D4 | 0.41(0.22,0.55) | 0.23(0.41,0.55) | 0.23(0.17,0.26) | 0.19(0.17,0.24) | 2.447 | 0.324 |  |
| C14:1 | 0.05(0.04,0.08) | 0.05(0.04,0.07) | 0.06(0.04,0.07) | 0.05(0.04,0.06) | 2.478 | 0.431 |  |
| C14DC | 0.02(0.00,0.02) | 0.02(0.00,0.02) | 0.02(0.01,0.02) | 0.01(0.01,0.02) | 3.275 | 0.660 |  |
| C14-OH | 0.03(0.02,0.04) | 0.02(0.02,0.03) | 0.02(0.02,0.03) | 0.02(0.02,0.03) | 2.179 | 0.062 |  |
| C12 | 0.04(0.03,0.05) | 0.04(0.03,0.06) | 0.05(0.04,0.07) | 0.04(0.02,0.04) | 3.611 | 0.055 |  |
| C10 | 0.06(0.04,0.08) | 0.05(0.04,0.07) | 0.05(0.04,0.07) | 0.05(0.04,0.07) | 2.378 | 0.824 |  |
| C26 | 0.03(0.02,0.04) | 0.02(0.02,0.03) | 0.02(0.02,0.03) | 0.02(0.02,0.03) | 3.225 | 0.195 |  |
| C24 | 0.04(0.02,0.05) | 0.03,0.02,0.04) | 0.03(0.03,0.04) | 0.03(0.03,0.04) | 2.173 | 0.166 |  |
| C22 | 0.05(0.03,0.07) | 0.03(0.03,0.06) | 0.05(0.03,0.06) | 0.03(0.03,0.05) | 2.678 | 0.051 |  |
| C20 | 0.02(0.01,0.02) | 0.02(0.01,0.02) | 0.01(0.01,0.02) | 0.01(0.01,0.02) | 3.275 | 0.127 |  |
| C18 | 0.45(0.39,0.55) | 0.38(0.32,0.47) | 0.40(0.28,0.54) | 0.45(0.40,0.45) | 2.179 | 0.139 |  |
| C16:1-OH | 0.04(0.03,0.05) | 0.03(0.03,0.05) | 0.04(0.03,0.05) | 0.04(0.03,0.05) | 2.673 | 0.734 |  |
| C16-OH | 0.03(0.02,0.03) | 0.03(0.02,0.04) | 0.03(0.02,0.05) | 0.02(0.02,0.03) | 3.125 | 0.263 |  |
| C4/C2 | 0.02(0.01,0.02) | 0.02(0.02,0.03) | 0.02(0.02,0.03) | 0.03(0.02,0.03) | 2.421 | 0.255 |  |
| C3/C16 | 1.85(1.63,2.45) | 2.02(1.69,2.80) | 2.11(1.48,2.76) | 1.34(0.95,2.75) | 3.225 | 0.627 |  |
| C3/C2 | 0.16(0.12,0.18) | 0.14,00.12,0.18) | 0.15(0.11,0.22) | 0.18(0.14,0.19) | 2.179 | 0.885 |  |
| C3/C0 | 0.43(0.34,0.57) | 0.41(0.33,0.52) | 0.37(0.30,0.48) | 0..36(0.29,0.39) | 3.611 | 0.128 |  |
| C2/C0 | 0.29(0.25,0.33) | 0.28(0.24,0.34) | 0.25(0.22,0.29) | 0.19(0.17,0.26) | 2.373 | 0.056 |  |
| C5-OH/C0 | 0.01(0.00,0.01) | 0.00(0.00,0.01) | 0.01(0.00,0.01) | 0.01(0.00,0.01) | 1.343 | 0.061 |  |
| C5-OH/C8 | 2.37(1.56,3.69) | 2.51(1.54,3.34) | 2.30(1.15,3.40) | 4.58(3.30,6.00) | 2.923 | 0.060 |  |
| C5/C3 | 0.07(0.06,0.10) | 0.08(0.05,0.11) | 0.08(0.06,0.12) | 0.09(0.08,0.12) | 1.924 | 0.272 |  |
| C5/C2 | 0.12(0.01,0.02) | 0.01(0.01,0.01) | 0.01(0.01,0.12) | 0.02(0.01,002) | 2.487 | 0.163 |  |
| C5/C0 | 0.00(0.00,0.01) | 0.00(0.00,0..00) | 0.00(0.00,0.00) | 0.00(0.00,0.00) | 2.643 | 0.173 |  |
| C4/C8 | 2.70(1.74,4.18) | 3.01(1.83,4.28) | 2.51(1.70,4.05) | 3.51(3.40,3.80) | 3.265 | 0.657 |  |
| C4/C3 | 0.13(0.09,0.17) | 0.14(0.09,0.18) | 0.13(0.10,0.21) | 0.14(0.13,0.18) | 2.179 | 0.600 |  |
| C14:1/C16 | 0.08(0.05,0.11) | 0.08(0.06,0.10) | 0.08(0.06,0.12) | 0.07(0.06,0.17) | 3.119 | 0.441 |  |
| C26/C20 | 1.60(1.07(2.66) | 1.53(1.31,2.63) | 1.97(1.31,2.63) | 1.31(1.07,2.63) | 3.420 | 0.992 |  |
| C16-OH/C16 | 0.04(0.03,0.05) | 0.04(0.03,0.06) | 0.05(0.03,0.07) | 0.03(0.03,0.04) | 1.142 | 0.086 |  |
| C8/C10 | 1.65(1.02,1.75) | 1.31(1.05,1.50) | 1.75(0.87,2.33) | 0.88(0.83,1.17) | 2.978 | 0.069 |  |
| C8/C2 | 0.01(0.01,0.01) | 0.01(0.01,0.01) | 0.01(0.01,0.01) | 0.01(0.01,0.01) | 1.585 | 0.940 |  |
| C5DC/C16 | 0.07(0.05,0.10) | 0.10(0.06,0.14) | 0.10(0.05,0.15) | 0.09(0.04,0.10) | 2.447 | 0.126 |  |
| C5DC/C5-OH | 0.23(0.19,0.40) | 0.45(0.30,0.70) | 0.42(0.20,0.67) | 0.20(0.13,0.26) | 2.678 | 0.068 |  |
| C10:2 | 0.56(0.41,0.80) | 0.50(0.37,0.84) | 0.48(0.38,1.00) | 0.50(0.39,0.63) | 3.275 | 0.947 |  |
| C10:1 | 0.07(0.04,0.10) | 0.06(0.04,0.10) | 0.05(0.04,0.07) | 0.06(0.05,0.08) | 2.179 | 0.341 |  |
| C18:1-OH | 0.02(0.01,0.02) | 0.02(0.01,0.02) | 0.02(0.01,0.02) | 0.01(0.01,0.03) | 3.111 | 0.387 |  |
| C18-OH | 0.02(0.01,0.02) | 0.01(0.01,0.02) | 0.01(0.01,0.02) | 0.01(0.01,0.02) | 3.854 | 0.501 |  |
| C18:1 | 0.44(0.38,0.56) | 0.41(0.32,0.52) | 0.41(0.28,0.48) | 0.35(0.29,0.43) | 1.234 | 0.115 |  |
| C3DC/C10 | 0.89(0.50,1.33) | 0.71(0.40,0.94) | 0.94(0.71,1.41) | 0.47(0.47,1.06) | 2.467 | 0.089 |  |
| C3DC | 0.05(0.03,0.07) | 0.04(0.02,0.05) | 0.05(0.03,0.06) | 0.03(0.02,0.06) | 2.248 | 0.127 |  |
| C5DC/C8 | 0.74(0.47,0.97) | 0.87(0.56,1.48) | 0.89(0.49,1.48) | 0.74(0.49,1.48) | 3.565 | 0.474 |  |
| C6DC | 0.32(0.27,0.49) | 0.27(0.24,0.36) | 0.26(0.23,0.28) | 0.26(0.25,0.27) | 2.239 | 0.058 |  |
| C10:2/C10 | 9.39(5.78,14.91) | 9.94(7.45,14.95) | 14.87(9.92,17.03) | 9.92(7.45,14.95) | 3.541 | 0.318 |  |
| C18:2 | 1.32(0.91,1.54) | 0.99(0.02,1.28) | 1.05(0.86,1.26) | 0.87(0.69,0.91) | 3.544 | 0.071 |  |
| C14:2 | 0.32(0.24,0.50) | 0.32(0.24,0.37) | 0.31(0.30,0.36) | 0.31(0.30,0.37) | 1.654 | 0.940 |  |
| C6  *P<0.05 | 0.75±0.36 | 0.71±0.25 | 0.70±0.25 | 0.60±0.30 | 0.844 | 0.473 |  |
